# Development of PP Compound Recipes Using Genetic Algorithms and Analytical Models

**DOI:** 10.3390/polym17081059

**Published:** 2025-04-14

**Authors:** Lukas Seifert, Lisa Leuchtenberger-Engel, Christian Hopmann

**Affiliations:** Institute for Plastics Processing (IKV) in Industry and Craft at RWTH Aachen University, Seffenter Weg 201, 52074 Aachen, Germany

**Keywords:** polypropylene compounds, genetic algorithms, analytical models, material optimisation, shear viscosity, tensile modulus, impact strength, injection moulding, packaging applications, polymer engineering

## Abstract

This study explores the development of polypropylene (PP) compound recipes using analytical models (AM) combined with genetic algorithms (GAs). A talcum-filled PP compound, commonly utilised in injection moulding for packaging applications, served as a reference material, with shear viscosity, tensile modulus, and impact strength selected as target properties for replication. The AM were adapted and fitted to a dataset of 52 compounds, achieving high predictive accuracy for shear viscosity and tensile modulus, while impact strength proved more challenging due to its inherent variability. Three recipes were generated using GA under predefined material constraints. Recipe 1 aimed to replicate all three target properties, achieving a balanced compromise with maximum deviations of 13.14% for tensile modulus and 12.37% for impact strength while closely matching shear viscosity (maximum 9.8% deviation). Recipes 2 and 3, focused solely on matching shear viscosity and impact strength, demonstrated exceptional accuracy for shear viscosity, with Recipe 2 achieving near-perfect alignment (2.5% deviation). However, neither recipe approached the tensile modulus target due to material limitations. The findings demonstrate the effectiveness of combining AM with GA for designing alternative formulations, emphasising the importance of realistic targets and material constraints. This methodology is highly adaptable, allowing for the inclusion of additional optimisation criteria such as cost or sustainability. Future work will explore broader material sets and properties, extending the framework’s applicability to technical polymers and diverse industrial applications.

## 1. Introduction

Polypropylene (PP) is one of the most widely used plastics in the world due to its relative ease of processing and balanced mechanical properties. As a result, PP is used in a wide range of applications, including packaging, construction, and the automotive industry [1]. Depending on the specific application, many properties of PP need to be optimised at the same time [2,3,4,5].

One of the key mechanical properties that determines the performance of PP is the tensile modulus [2,6,7,8,9]. Defined as the relationship between tensile stress and strain, tensile modulus indicates the stiffness of a material and is therefore essential in applications where structural integrity is paramount [10]. In packaging, for example, the tensile modulus directly affects a container’s ability to protect its contents under mechanical stresses such as those encountered during stacking, transport, or handling. A higher tensile modulus helps prevent undesirable deformation, while keeping the packaging lightweight and cost effective [9]. Achieving this balance is critical to developing packaging solutions that meet the stringent demands of today’s markets.

While tensile modulus is primarily concerned with the ability of the material to withstand slowly applied or static forces, impact strength of PP is concerned with its ability to withstand sudden, high speed forces [9,11]. In packaging applications, impact strength becomes important when containers are dropped; robust impact strength ensures that the packaging remains intact, protecting the goods inside. Similarly, impact strength is a key requirement in automotive components, such as bumpers, where sudden forces may occur in accidents, and in construction applications, such as PP pipes that must withstand the rigours of on-site handling [9].

In addition to these mechanical properties, viscosity is of paramount importance when processing PP parts by injection moulding or extrusion [12,13,14,15]. In particular, shear viscosity plays a critical role in ensuring consistent cavity filling during injection moulding; if it is too high, the cavity may not be completely filled, while too low a viscosity may lead to leakage or overfilling [14,15]. A similar concern applies to extrusion, where maintaining a stable and appropriate viscosity is essential to minimise material waste and ensure a reliable manufacturing process [13].

Balancing the desired mechanical and processing properties often requires the development of specially tailored compound formulations [2,3,4,8,16]. During the compounding process, different grades of PP are blended with additives and fillers to achieve a specific set of properties. However, as more components are introduced, the complexity of the formulation increases significantly due to potential non-linear interactions between the components. This complexity presents a multi-dimensional optimisation challenge, requiring an effective and systematic recipe development strategy to identify formulations that meet both manufacturing and end-use requirements.

## 2. Development of New Recipes Using Analytical Models and Genetic Algorithms

The traditional development of polymer compound formulations has long relied on the expertise and intuition of experienced compound formulators. This iterative process, while invaluable, is inherently time-intensive and resource-demanding. Adjustments to recipes are often achieved through trial and error, a method ill-suited for handling the increasing complexity of modern formulations, particularly those involving multiple additives or recycled components. For simple PP mixtures containing only various types of polymers, mixing rules can be applied to systematically predict the properties of the final compound [2,17,18,19]. Mixing rules predict the compound properties fairly precise and can be applied with just a few existing test points. However, when additives or fillers are introduced, simple mixing rules cannot be applied anymore. For the interaction between the individual fillers, additives, and polymers, several mixing rules would have to be applied, but there is currently no systematic procedure for combining these with each other. Therefore, to predict the properties of more complex formulations, machine learning methods (ML) such as Artificial Neural Networks (ANNs) or Symbolic Regression methods (SR) are amongst the most prominent currently utilised approaches. These offer predictive accuracy for properties like tensile modulus, impact strength, and shear viscosity [17,20,21,22,23,24,25,26] and can be applied on arbitrarily complex compound formulations with numerous individual components. However, these methods require extensive datasets that document not only the material properties but also detailed processing conditions. The high cost and effort required to generate such datasets limit their adoption in many industrial contexts [27,28,29]. In addition, when applied to smaller datasets, machine learning methods are very sensitive to individual measurement outliers, which can strongly influence the overall prediction quality of the model.

### 2.1. Prediction of Compound Properties with Analytical Models

**Figure 1:**
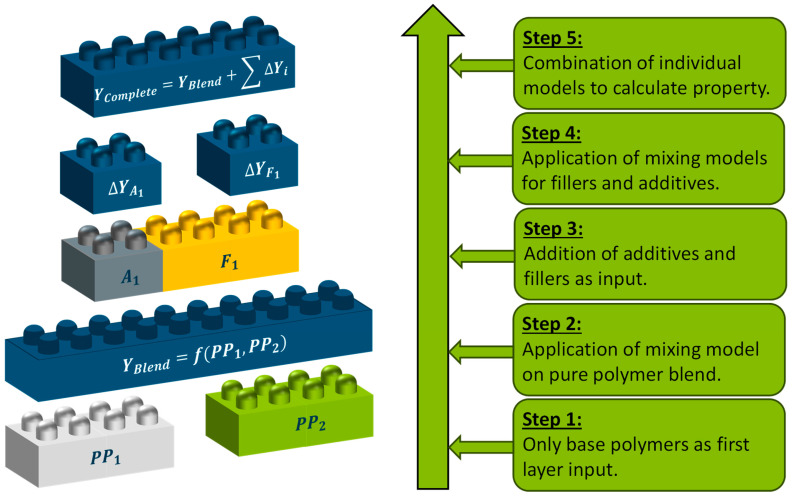
Basis principle of analytical models for compound property prediction.

In response to the limitations of machine learning (ML) methods, analytical models (AM) have emerged as a practical alternative. These models build on the wide range of simple mixing rules reported in the literature by incorporating simple mathematical expressions to describe the individual effects of different fillers or additives. The underlying principle to construct such an AM is shown in Figure 1. At first, only the base polymers (e.g., PP_1_ and PP_2_) are applied on a basic mixing rule to predict the target property Y_Blend_. Afterwards, the effect of each introduced additive or filler is modelled separately for the mixture of a polymer with the base property Y_Blend_ and the filler or additive. In a final step, all individual impacts of fillers, additives, and polymers are combined to calculate the desired property Y_Complete_.

Analytical models have already been developed for predicting both tensile modulus and shear viscosity, achieving coefficients of determination (R^2^) as high as 0.98 for shear viscosity and 0.97 for tensile modulus [8,16]. Notably, these results were obtained for compounds containing two types of PP, two types of filler, and two additives. While comparable approaches typically rely on thousands of fitting parameters to achieve similar levels of predictive accuracy, AM require only about 10–15 fitting parameters [8,16]. This relatively small number of parameters allows convenient adaptation to different processing conditions, even when only a limited amount of data is available. Furthermore, AM can be easily adapted to new datasets, making them particularly well suited to contexts where data may be scarce or incomplete [30].

### 2.2. Development of New Recipes Using Analytical Models

The prediction model for determining the compound properties as a function of the formulation is the first necessary component for formulation development. In a second step, the individual formulation compositions must be systematically varied in order to achieve the target specifications according to the predictions with the model. In this paper, previously developed AM [8,16] are used as the prediction model and genetic algorithms (GAs) are used for the optimisation. By characterising a target material in terms of tensile modulus, impact strength, and shear viscosity profile, new formulations can be derived by integrating AM for property prediction and GA for recipe optimisation. This approach directly addresses the challenge of balancing multiple properties within specific material and process constraints. Compared to traditional or purely ML-based methods, the proposed framework is computationally efficient, adaptable, and scalable, providing a robust solution for modern recipe development in the polymer industry. Furthermore, due to the underlying principle of the AM, they can be easily adapted to be used on different processing machines and are applicable on smaller datasets compared to ML approaches.

**Figure 2:**
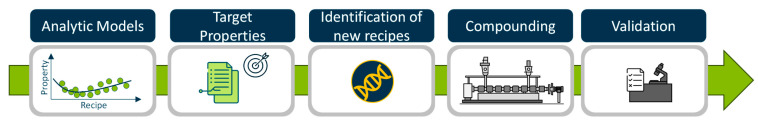
Schematic representation of methodology in this publication.

The approach of this paper is illustrated in Figure 2.

Firstly, the AM for predicting impact strength, shear viscosity, and tensile modulus are derived from existing experimental data. Secondly, target properties are selected for the identification of new formulations. Using GA, three new recipes are identified and produced in practical compounding trials. For each identified formulation, the mechanical and rheological properties are determined in the laboratory and compared with the target properties for validation.

## 3. Materials and Methods

In the following, the materials, the characterisation methods for determining the investigated compound properties, and the laboratory equipment used for compounding are introduced and explained.

### 3.1. Materials and Characterisation

As a target material for the development of new recipes, a material by SÜDPACK Verpackungen GmbH, Ochsenhausen, Germany, is chosen. The material StarBlend^®^ PP 012-01 R T20 N utilises bio-based PP, which contains talcum as a filler that modifies the impact strength of the material. It is commonly used in injection moulding for packaging applications. The materials chosen for the identification of new recipes are identical with the materials used in previous investigations for the creation of the AM [8,16]. As base polymers, two homopolymer PP grades 505P by Saudi Basic Industries Corporation (SABIC) (Riyadh, Saudi Arabia) and HP548R by LyondellBasell (Rotterdam, The Netherlands) were used [31,32]. To specifically adjust the tensile modulus, chalk Omyalite 50 H supplied by OMYA GmbH (Oftringen, Switzerland) was used. For the modification of viscosity and impact strength, the additives Polyvel CR5P by Polyvel Europe GmbH (Jork, Germany) and Engage 8200 by DOW Inc. (Midland, MI, USA) were used [33,34].

To specify the target compound properties for replication, the shear viscosity, tensile modulus, and impact strength of the target material were determined. The properties to be reproduced are given in Table 1 and Table 2.

### 3.2. Laboratory Equipment for Compounding

The compounding trials were conducted on a co-rotating twin screw extruder (Coperion GmbH, Stuttgart, Germany) with a screw diameter of 26 mm. Similarly to the previous trials [8,16] for the creation of a dataset for the development of the AM, all compounding trials were conducted with a compounder speed of 300 min^−1^, a housing temperature of 210 °C, and a throughput of 15 kg/h [8,16]. The screw elements consisted of conveying elements with a combination of kneading and mixing elements at the beginning of the process to plasticise the polymers.

The shear viscosity of the compounds was measured at a temperature of 230 °C using three round capillaries (diameter 1 mm) with lengths of 20 mm, 10 mm, and 5 mm on a high-pressure capillary rheometer (RHEOGRAPH 50, GÖTTFERT Werkstoff-Prüfmaschinen GmbH, Buchen, Germany). To characterise the tensile modulus, type 1A specimens were produced in accordance with DIN EN ISO 527 on an IntElect 100–250 injection moulding machine from Sumitomo (SHI) Demag Plastics Machinery GmbH (Schwaig, Germany) [35]. The specimens were tested on a Z100 tensile testing machine manufactured by ZwickRoell GmbH & Co. KG, Ulm, Germany. A test speed of 1 mm/min was used to determine the tensile modulus in accordance with DIN EN ISO 527 [35]. For the determination of the impact strength, the same type 1A specimens were used. They were cut to the specimen geometry of 80 ± 2 mm in length and 10 ± 0,2 mm in width. Additionally, the test specimens were notched and tested according to DIN EN ISO 179-1eA [36]. For both tensile modulus and impact strength, a minimum of 10 specimen were tested and used for the determination of the average tensile modulus and average impact strength.

## 4. Fitting the Models on the Dataset

For the development of the AM in the previous trials [8,16], the same base polymers as wells as additives and fillers were used. However, contrary to this investigation, a second type of chalk was present in the dataset besides Omyalite 50 H. Therefore, the previously developed AM [8,16] were simplified by removing the partial model for the second type of chalk and fitting the remaining parameters again.

The parameter fitting was carried out using the Python library scikit-learn (sklearn) version 1.3.1 on measurement data obtained from a total of 52 compound formulations [37]. To achieve the best fit, the mean squared error (MSE) between the model predictions and experimental data were minimised. The resulting model equations and corresponding coefficients are provided in Appendix A. Similarly to the development of the AM for predicting the tensile modulus $E_{mix,\;compound}$ and the shear viscosity $\eta_{mix}(\dot{\gamma })$, a third AM was identified for the prediction of the impact strength $W_{mix,\;compound}$. The details of this model and its coefficients can also be found in Appendix A.

To assess the accuracy of all three models, both the mean absolute error (MAE) and the coefficient of determination (R^2^) were calculated. The R^2^ value measures the proportion of variance in the observed data explained by the model, ranging from 0 to 1, with higher values indicating a better fit. An R^2^ value below 0 signifies that the model performs worse than a simple horizontal line representing the mean of the data.

Table 3 presents the MAE and R^2^ values for all three models. The highest prediction accuracy was achieved for shear viscosity and tensile modulus. Specifically, the shear viscosity model reached an R^2^ of 0.9904 and an MAE of 7.696. For tensile modulus, the R^2^ of 0.9709 is slightly lower, but the associated MAE of 18.279 N/mm^2^ is still below the average experimental deviation of 25.131 N/mm^2^ observed in the 52 compounds. In contrast, the impact strength model demonstrated a lower performance, with an R^2^ of 0.9196 and an MAE of 0.218. During impact strength testing, the average measurement deviation was approximately 0.456 kJ/m^2^, representing around 12% of the measured impact strength. Based on this dataset, it was therefore to be expected that the impact strength AM would not achieve the same level of accuracy as the shear viscosity and tensile modulus models. Nonetheless, this AM for impact strength will be employed alongside the other two models in the subsequent development of alternative compound formulations.

## 5. Generating New Recipes with GA

**Figure 3:**
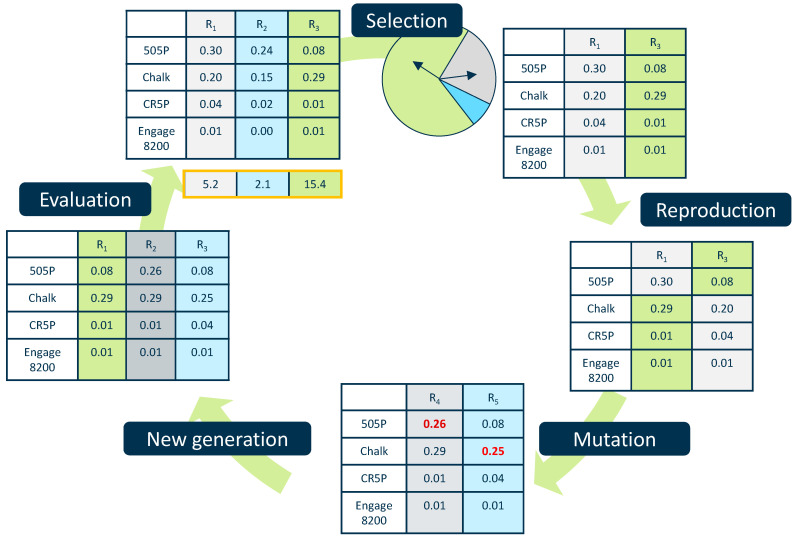
Principle GA for generation of compound formulations. The red numbers indicate a change from the previous step.

For the implementation of GA, the Python framework PyGAD version 3.4.0 is used to identify formulations fullfiling the specified material properties given in Table 1 [38]. The underlying principle of finding new formulations using GA is illustrated in Figure 3.

At the start of the GA, an initial population of multiple individuals is randomly generated. Each individual, labelled as R_1_, R_2_, and so on, represents a potential recipe formulation and comprises several chromosomes. In this study, the chromosomes denote the proportions of 505P, chalk, CR5P (peroxide additive), and Engage 8200 (impact modifier). Once the initial population is established, the fitness of each individual is assessed. In the context of this paper, fitness indicates how well the predicted properties—shear viscosity, impact strength, and tensile modulus—match the target values according to the AM. A higher fitness value signifies a better solution with less deviation to the target.

From the initial population, the subset of individuals with the highest fitness values is selected. During subsequent evolutionary steps, these selected individuals may recombine to form new individuals, and the chromosomes (i.e., proportions of specific components) may mutate at random. Finally, from the pool of original, recombined, and mutated individuals, a new generation is formed and evaluated. This process repeats until a designated termination criterion is reached or a predefined number of generations is completed.

To identify new recipes, a set of boundaries was imposed for each component. A maximum of 30% chalk was specified, mirroring the upper limit used in the previous compounding trials and model training [8,16], and a minimum of 5% was enforced to accommodate the throughput constraints of the dosing equipment. For Engage 8200, the maximum content was set at 4%, while for Polyvel CR5P, it was capped at 1%, reflecting the upper limits from the initial model training experiments [8,16].

Furthermore, the total share of all components had to sum to 100%. To ensure this condition, the GA varied the proportions of 505P along with all additives and fillers, and the share of HP548R was then calculated so that the total reached 100%. Table 4 summarises the main parameters for the GA in the PyGAD implementation.

In order to replicate the reference material, three recipes were generated, each based on a different fitness function. The optimisation criteria for the identification of new compound formulations can be arbitrarily complex and depend on the application (e.g., including price as a factor or CO_2_ footprint). The purpose of this research is to demonstrate the general applicability of the combination of AM and GA. The first recipe was optimised to simultaneously match all six target shear viscosities as well as the tensile modulus and impact strength, assigning equal importance to each property. Since the magnitude of the desired impact strength (~1) differs from that of the tensile modulus (~1000), the fitness function was constructed around the minimum percentage deviation between the model-predicted properties and the target values. This fitness function, which is maximised during GA iterations, is presented in Equation (1).

(1) $$fitness=\frac{1}{\sum PE+10^{-6}}$$

Here, PE are the individual percentage errors calculated for each property and summed up. A small constant of $10^{-6}$ is added to prevent division by zero. Under these constraints, nearly all optimised solutions utilised the maximum chalk level of 30%. However, according to the AM’s predictions, this achieved only about 2200 N/mm^2^, falling short of the desired 2492.7 N/mm^2^ tensile modulus. It follows that increasing the chalk limit would be necessary to reach the target tensile modulus.

**Figure 4:**
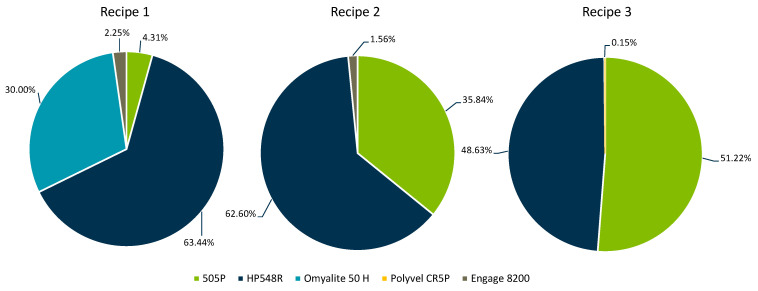
The shares of the individual recipe components for the three formulations identified using GA.

For the second and third recipes, the objective was to match the shear viscosities and impact strength while disregarding the tensile modulus. In the second recipe, the same fitness function was employed, summing all individual percentage errors for the six shear viscosity targets and the impact strength. In the third recipe, the fitness function was modified by first averaging the six individual shear viscosity errors before including the impact strength deviation. Ultimately, three alternative recipes were identified, as depicted in Figure 4, and subsequently processed.

## 6. Practical Validation of the Identified Recipes

The three identified recipes were compounded using a process setup and parameters similar to those employed for the compounds used in developing the AM. In the following subsections, the performance of these recipes is evaluated with respect to the three key compound properties.

### 6.1. Shear Viscosity

**Figure 5:**
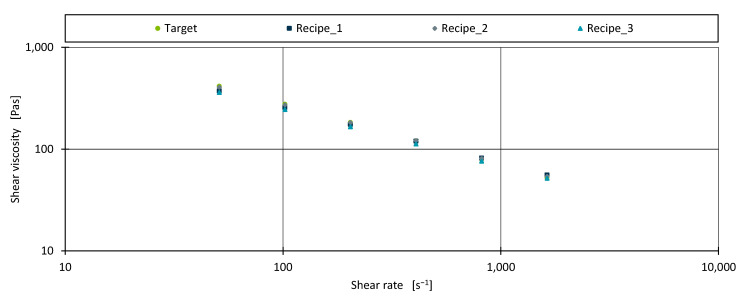
The shear viscosity for the tested shear rates for the target material and the three identified recipes.

Figure 5 compares the measured shear viscosities of both the target material and the three identified recipes.

Overall, the new formulations align well with the target shear viscosities, although higher deviations can be seen at lower shear rates, where absolute shear viscosities are typically greater. Table 5 summarises the absolute percentage deviations of each recipe relative to the target material.

Recipe 1 and Recipe 3 exhibit the highest percentage deviations, peaking at the lowest shear rate (51 s^−1^) with a deviation of 12.853% for Recipe 3 and 9.789% for Recipe 1. By contrast, Recipe 2 demonstrates particularly low percentage errors, with its largest deviation being 2.464% at 1632 s^−1^.

One reason for these discrepancies may be variations in how accurately the AM predicts the effects of different fillers and additives. In developing the AM for shear viscosity, the impact modifier was found to have a negligible effect, which simplifies modelling for a blend of the two base polymers with a small amount of impact modifier. Conversely, the inclusion of chalk in Recipe 1 or the non-linear influence of the peroxide additive in Recipe 3 increases model complexity and may result in higher prediction errors. However, because only three recipes were evaluated, caution should be exercised when drawing conclusions; it is possible that the strong performance of Recipe 2 could partly reflect a fortunate combination of ingredients.

### 6.2. Tensile Modulus

**Figure 6:**
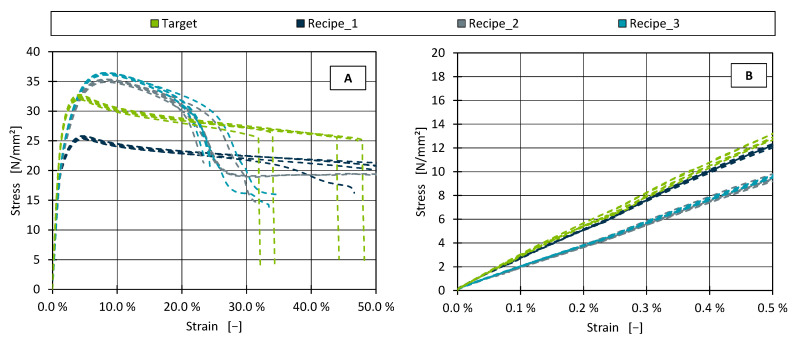
Stress–strain curves for the target material and the identified recipes for the full test (**A**) and the strain range for the determination of the tensile modulus (**B**).

Figure 6 shows the stress–strain curves of the three identified formulations and the target material.

**Figure 7:**
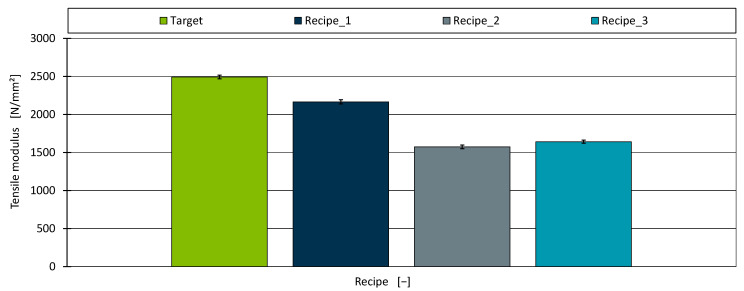
The tensile modulus for the target material and the three identified recipes.

Looking at the overall stress–strain curve (A), it is initially noticeable that the curves of all three replicated formulations differ significantly from the target material in the range up to 50% strain and diverge at the yield stress. However, the aim of identifying alternative formulations is only to simulate the modulus of elasticity. For this purpose, the strain range relevant to the determination is shown in Figure 6B. According to the standard, the tensile modulus is determined in the range 0.05% to 0.25% strain. It is immediately apparent that Recipe 1 and the target are relatively congruent, and Recipe 2 and Recipe 3 are flatter. The modulus of elasticity determined from these curves according to the standard is shown in Figure 7. None of the identified recipes managed to reach the target tensile modulus of 2492.7 N/mm^2^.

As detailed previously, the highest tensile modulus in the dataset used to develop the AM was approximately 2010 N/mm^2^. Therefore, only Recipe 1 aimed to approach the target tensile modulus, while Recipe 2 and Recipe 3 disregarded this property. As is consistent with that objective, Recipe 1 reached 2165.12 N/mm^2^ by incorporating 30% chalk, whereas Recipe 2 and Recipe 3—both of which omitted chalk—achieved only 1574.2 N/mm^2^ and 1641.6 N/mm^2^, respectively. Accordingly, the percentage deviations from the target tensile modulus were 13.14% for Recipe 1, 36.85% for Recipe 2, and 34.15% for Recipe 3.

Although these deviations are considerably larger than those observed for shear viscosity, they do not necessarily reflect poor model performance. Rather, they indicate that, with the available materials, it was not possible to replicate the target tensile modulus. Had the desired modulus been lower, closer alignment would have been feasible.

### 6.3. Impact Strength

**Figure 8:**
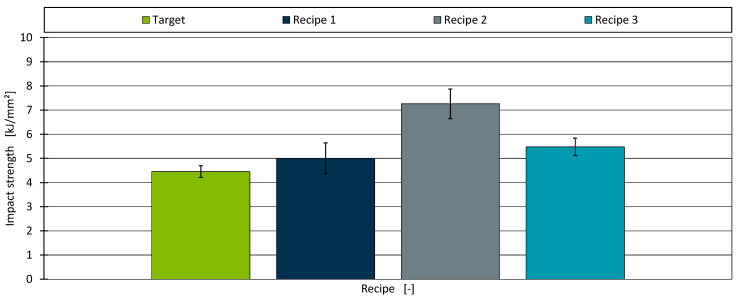
The impact strength for the target material and the three identified recipes.

Finally, Figure 8 displays the impact strengths of the three identified recipes compared to the target value of 4.45 kJ/m^2^. All three new formulations exceeded this target.

Recipe 1 attained an impact strength of 5.00 kJ/m^2^, with a measurement uncertainty of 0.64 kJ/m^2^, placing it relatively close to the target. By contrast, Recipe 2 (incorporating 1.56% impact modifier) reached 7.26 kJ/m^2^, with a measurement deviation of 0.62 kJ/m^2^. This large discrepancy may be attributed to the overall predictive inaccuracy of the AM for impact strength whose average prediction error was 0.22 kJ/m^2^. Recipe 3 (5.48 kJ/m^2^) also surpassed the target, though with a smaller deviation than Recipe 2. Table 6 provides the absolute percentage deviations for all three recipes.

## 7. Discussion

This study explored the feasibility of using a previously developed AM framework in conjunction with genetic algorithms (GAs) to generate alternative PP compound formulations. The target was to replicate rheological and mechanical properties—specifically shear viscosity, impact strength, and tensile modulus—of a talcum-filled PP compound commonly applied in injection moulding for packaging.

The results highlight the potential of this approach, particularly in reproducing shear viscosity. Among the three optimised recipes, Recipe 2 achieved a near-exact match, showing only a 2.5% deviation from the target, whereas Recipes 1 and 3 reached acceptable, albeit somewhat higher, deviations of up to 13%. These differences in shear viscosity performance align with the predictive limitations of the AM, especially for more complex formulations containing chalk or peroxide additives. Such fillers can introduce non-linear effects that are harder to model accurately.

The mechanical properties proved more challenging to replicate precisely. Recipe 1 was specifically optimised to approach the target tensile modulus, achieving a deviation of approximately 13%. In contrast, Recipe 2 and Recipe 3, which prioritised shear viscosity and impact strength alone, differed significantly from the target modulus. The shortfall in reaching the designated modulus underscores the inherent material limitations, particularly the 30% upper limit on chalk content. Impact strength predictions also varied substantially; while Recipe 1 remained within roughly 12% of the target, Recipe 2 overshot by more than 60%. These results show the difficulties of modelling impact strength accurately, given both the intrinsic variability of impact testing and potential batch-to-batch variations in the impact modifier.

Overall, Recipe 1 proved to be the most balanced formulation, providing a reasonable compromise for all three properties. Its inclusion of 30% chalk and an impact modifier enabled it to approach the target properties within the given material constraints. Recipes 2 and 3, which were tailored for shear viscosity and impact strength only, excelled at matching viscosity but lacked the compositional elements to approach tensile modulus.

## 8. Conclusions

A reference material typically used for injection moulding in packaging applications was selected and characterised for shear viscosity, tensile modulus, and impact strength. As a research question, the combination of AM and GA to replicate these properties was posed and answered in this paper.

In conclusion, this research confirms that AM can be successfully combined with GA to design new PP compound formulations under realistic processing and material constraints. The accuracy of the prediction is primarily determined by the predictive quality of the AM. The more accurately these models are able to map the complex interactions of the individual formulation components, the better the formulations identified by the GA will be. Throughout the paper, the proposed method reliably identified recipes that closely matched the desired shear viscosity, demonstrating its potential for identifying suitable formulations. Although the predictive accuracy for tensile modulus and impact strength proved more challenging, the observed deviations largely reflect the limited breadth of the available materials and the intrinsic variability of mechanical testing, rather than a fundamental flaw in the modelling framework.

Moreover, the approach allows for straightforward incorporation of additional considerations—such as cost or environmental impact—into the optimisation process, thereby improving its usefulness for industrial applications. By expanding the range of materials, enhancing the scope of target properties, and integrating uncertainty quantification, future studies can further refine the precision, scalability, and industrial relevance of this computational framework.

## Figures and Tables

**Table 1:** The shear viscosity of the reference material for the recreation of new recipes.

Shear rate [s^−1^]	51	102	204	408	816	1632
Shear viscosity [Pas]	413.7	275.6	182.6	120.7	79.7	52.6

**Table 2:** The tensile modulus and impact strength of the reference material for the recreation of new recipes.

Property	Average	Standard Deviation
Tensile modulus	2492.7 N/mm^2^	24.08 N/mm^2^
Impact strength	4.45 kJ/m^2^	0.24 kJ/m^2^

**Table 3:** The achieved R^2^ and MAE values for the AM fitted on the training dataset.

Prediction	R^2^	MAE
Shear viscosity	0.9904	7.696
Tensile modulus	0.9709	18.279
Impact strength	0.9196	0.218

**Table 4:** Settings for GA using PyGAD.

Parameter	Value/Setting
Number of generations	1000
Number of parents	20
Size of population	100
Genes	4
Mutation type	random
Mutation chance	20%
Crossover type	single_point

**Table 5:** The absolute percentage deviations between the shear viscosity of the identified and compounded recipes compared to the target shear viscosities.

Shear Rate [s^−1^]	Percentage Deviation in Shear Viscosity Compared to Target [%]		
	Recipe 1	Recipe 2	Recipe 3
51	9.789	1.890	12.853
102	7.109	1.111	11.308
204	4.136	0.264	9.276
408	0.956	0.624	6.955
816	2.390	1.536	4.451
1632	5.880	2.464	1.815

**Table 6:** The absolute percentage deviations between the impact strength of the identified and compounded recipes compared to the target impact strength.

Recipe	Absolute Percentage Deviation [%]
Recipe 1	12.37
Recipe 2	62.96
Recipe 3	22.97

## Data Availability

The original contributions presented in this study are included in the article. Further inquiries can be directed to the corresponding author.

## Appendix A

### Appendix A.1. An Analytical Model for the Prediction of the Tensile Modulus

(A1)
$$E_{mix,\;compound}=E_{mix,\;blend}+{\Delta E}_{mix,\;chalk}+{\Delta E}_{mix,impact}+{\Delta E}_{mix,peroxide}$$

(A2)
$$E_{mix,\;blend}=\left|E_{HP548}-E_{505P}\right|\times a\times \ln\left(\frac{E_{\mathrm{HP}548}}{E_{505P}}\times b\times \frac{x_{HP548}}{x_{505P}+x_{HP548R}}+\frac{E_{HP548}}{E_{505P}}\times c\right)+E_{505P}\times \;d$$

(A3)
$$\;{\Delta E}_{mix,\;chalk}=x_{Chalk}\times (I_{Chalk}+E_{mix,\;blend}\times s_{Chalk})$$

(A4)
$${\Delta E}_{mix,impact}=x_{Impact}\times (I_{Impact}+E_{mix,\;blend}\times s_{Impact})$$

(A5)
$${\Delta E}_{mix,peroxide}=x_{Peroxide}\times (p_{1}{\times \left(100\times \frac{x_{505P}}{x_{505P}+x_{HP548R}}\right)}^{p_{2}})$$

### Appendix A.2. Model Coefficients for the AM for the Prediction of the Tensile Modulus

**Table A1:** Model parameters for the AM for the prediction of the tensile modulus.

Parameter	Value	Parameter	Value
a	0.28412	$p_{1}$	−0.0001
b	8.15142	$I_{Chalk}$	8203.428
c	0.62736	$I_{Impact}$	4761.649
d	1.02487	$s_{Chalk}$	−4.166
$E_{505P}$	1388.14	$s_{Impact}$	−4.565
$E_{HP548}$	1631.38	$p_{2}$	4.002

### Appendix A.3. An Analytical Model for the Prediction of Shear Viscosity

(A6)
$$\eta_{mix}(\dot{\gamma })=\eta_{mix,\;blend}+∆\eta_{Peroxide}+∆\eta_{Chalk}$$

(A7)
$$\eta_{mix,\;blend}\left(\dot{\gamma }\right)=x_{505P}\eta_{505P}\left(\dot{\gamma }\right)+x_{HP548R}\eta_{HP548R}\left(\dot{\gamma }\right)$$

(A8)
$$\eta \left(\dot{\gamma }\right)=\frac{A}{{\left(1+B\dot{\gamma }\right)}^{C}}$$

(A9)
$$∆\eta_{Peroxide}=\eta_{mix,\;blend}\left(\dot{\gamma }\right)\times e^{x_{Peroxide}\times (p_{3}\times \ln\left(\dot{\gamma }\right)+p_{4})}-\eta_{mix,\;blend}\left(\dot{\gamma }\right)$$

(A10)
$$∆\eta_{Chalk}=x_{Chalk}\times (c_{1}\times \eta_{mix,\;blend}\left(\dot{\gamma }\right)-c_{2})$$

### Appendix A.4. Model Coefficients for the AM for the Prediction of Shear Viscosity

**Table A2:** Model parameters for the AM for the prediction of shear viscosity.

Parameter	Value	Parameter	Value
$A_{505P}$	400.022	$C_{HP548R}$	0.69034
$B_{505P}$	0.00144	$p_{3}$	19.946
$C_{505P}$	0.66601	$p_{4}$	−188.420
$A_{HP548R}$	2513.790	$c_{1}$	0.650
$B_{HP548R}$	0.01328	$c_{2}$	19.027

### Appendix A.5. An Analytical Model for the Prediction of Impact Strength

(A11)
$$W_{mix,\;compound}=W_{mix,\;blend}+{\Delta W}_{mix,\;chalk}+{\Delta W}_{mix,impact}+{\Delta W}_{mix,peroxide}$$

(A12)
$$W_{mix,\;blend}=\frac{x_{505P}}{x_{505P}+x_{HP548R}}\times W_{505P}+\frac{x_{HP548}}{x_{505P}+x_{HP548R}}\times W_{HP548}$$

(A13)
$${\Delta W}_{mix,\;chalk}=x_{Chalk}\times \left(\frac{x_{505P}}{x_{505P}+x_{HP548R}}\times I_{Chalk,2}+s_{Chalk,2}\right)$$

(A14)
$${\Delta W}_{mix,\;impact}=x_{Impact}\times \left(\frac{x_{505P}}{x_{505P}+x_{HP548R}}\times I_{Impact,2}+s_{Impact,2}\right)$$

(A15)
$${\Delta W}_{mix,\;peroxide}=x_{Peroxide}\times \left(\frac{x_{505P}}{x_{505P}+x_{HP548R}}\times I_{Peroxide}+s_{Peroxide}\right)$$

### Appendix A.6. Model Coefficients for the AM for the Prediction of Impact Strength

**Table A3:** Model parameters for the AM for the prediction of the impact strength.

Parameter	Value	Parameter	Value
$W_{505P}$	3.7129	$I_{Peroxide}$	−147.4393
$W_{HP548}$	3.7494	$s_{Chalk,2}$	1.8564
$I_{Chalk,2}$	−2.0589	$s_{Impact,2}$	−23.8065
$I_{Impact,2}$	60.7621	$s_{Peroxide}$	22.8145
